# Supplementary material for: A Comparative Study on Hexavalent Chromium Adsorption onto Chitosan and Chitosan-Based Composites
Source: Polymers (Basel). 2021 Oct 6;13(19):3427. doi: 10.3390/polym13193427 (PMC8512014; doi:10.3390/polym13193427)
Supplement: Supplementary file 1 [file polymers-13-03427-s001.zip › polymers-1372730-supplementary.pdf]

<u>**Supplementary material**</u>

A comparative study on hexavalent chromium adsorption onto chitosan and chitosan-based composites

Rachid El Kaim Billah[a], Moonis Ali Khan[b,*], Young-Kwon Park[c], Amira AM[d], Hicham Majdoubi[e], Younesse Haddaji[e], Byong-Hun Jeon[f]

[a]Department of Chemistry, University of Chouaib Doukkali, Faculty of Sciences, Laboratory of Coordination and Analytical Chemistry, Avenue Jabran Khalil Jabran B.P 299-24000, El Jadida, Morocco.

[b]Chemistry Department, College of Science, King Saud University, Riyadh 11451, Saudi Arabia.

[c]School of Environmental Engineering, University of Seoul, Seoul 02504, Republic of Korea

[d]Department of Chemistry, University of Hassan II-Casablanca, Faculty of Sciences Ben M'Sik, Laboratory of analytical chemistry and physico-chemistry of materials (Casablanca) Morocco.

[e]Laboratory of Engineering and Materials, Department of Chemistry, Faculty of Sciences Ben M'Sik, University of Hassan II-Casablanca, Casablanca 21100, Morocco

[f]Department of Earth Resources and Environmental Engineering, Hanyang University, Seoul 04763, Republic of Korea

*Corresponding Author: Moonis Ali Khan
E-mail addresses: mokhan@ksu.edu.sa; moonisalikhan@gmail.com

Table of content

Index	Caption
**Text S1**	Colorimetric method for Cr(VI) determination by UV-Visible Spectrometer
**Text S2**	Kinetics models
**Text S3**	Isotherm models
**Text S4**	Thermodynamics parameters
**Figure S1**	Linearized pseudo-first-order (a), and pseudo-second-order (b) kinetic plots for Cr(VI) adsorption
**Figure S2**	Linearized Langmuir **(a)**, and Freundlich **(b)** isotherm plots for Cr(VI) adsorption.
**Figure S3**	Van’t Hoff’s plots for Cr(VI) adsorption

Text S1. Colorimetric method for Cr(VI) determination by UV-visible spectrometer

The colorimetric method for Cr(VI) determination [29] was conducted following the guide lines of the NF T90-043 standard, France. 1, 5-diphenylcarbazide (DPC) solution was prepared by mixing 0.02 g of DPC with 10 mL of ethanol (100%) and 40 mL of sulfuric acid (1.8 M). Complete dissolution of the reagent was achieved after 1 day by heating at 60 °C or after 2 days without heating. The measurement of residual Cr(VI) concentrations was performed as follows: 1.2 mL of DPC solution and 0.1 mL of concentrated nitric acid were added to 20 mL of the sample. An intensely colored purple complex appears, originating from the redox reaction between DPC and Cr(VI). Cr(VI) was reduced to Cr(III) thereby forming a Cr(III)–diphenylcarbazone complex, which was color giving [29, 30]. However, this complex had a limited stability of about 15 min. Absorption was measured at 560 nm wavelength. Linearity between absorbance and concentration was verified in Cr(VI) concentration range of 0–0.5 mg/L.

Text S2. Adsorption kinetic models

The pseudo-first-order [40] and pseudo-second-order kinetic [41] models in linearized forms are respectively expressed by equations S1 and S2:

$$log(q_{e1} - q_t) = logq_{e1} - \frac{k_1}{2.303} \times t \qquad (S1)$$

$$\frac{t}{q_t} = \frac{1}{k_2 q_{e2}^2} + \frac{1}{q_{e2}} \times t \qquad (S2)$$

where q_t (mg/g) is the experimental adsorbed concentration at a time t; q_{e1} (mg/g) and q_{e2} (mg/g) are the adsorbed concentrations of MB at the equilibrium as calculated by the pseudo-first-order model and the pseudo-second-order kinetic models, respectively; k_1 (1/min) and k_2 (g/mg-min) are the pseudo-first-order and pseudo-second-order rate constants, respectively.

Text S3. Isotherm models

Langmuir isotherm model [42] assumes formation of monomolecular layer over adsorbent surface without interaction between the adsorbed molecules. Langmuir isotherm model in linearized form is expressed as:

$$\frac{C_e}{q_e} = \frac{1}{K_L q_m} + \frac{1}{q_m} \times C_e \qquad (S3)$$

where q_m (mg/g) and K_L (L/mg) are the constants for maximum monolayer adsorption capacity and a constant related to the heat of adsorption, respectively.

Freundlich isotherm model [43] in linear form is expressed as:

$$log\ q_e = logK_F + \frac{1}{n}\ logC_e \qquad (S4)$$

where K_F ((mg/g) (L/mg)$^{(1/n)}$) and n are the Freundlich constants related to bonding energy and deviation in adsorption from linearity, respectively. If n =1 (linear adsorption process), $n < 1$ (chemical adsorption process), $n > 1$ (physical adsorption process).

Text S4. Adsorption thermodynamic parameters

Thermodynamic parameters viz. standard Gibb's free energy change (ΔG°), entropy change (ΔS°), and enthalpy change (ΔH°) are calculated as:

$$\Delta G° = -RT\ ln\ K_c \qquad (S5)$$

$$ln\ K_c = \frac{\Delta S°}{R} - \frac{\Delta H°}{R} \times \frac{1}{T} \qquad (S6)$$

where R is the universal gas constant (8.314 J/mol-K), T is the absolute temperature (K), and K_c (dimensionless) is the separation factor $K_c = C_{Ae}/C_e$. ΔS° and ΔH° were calculated from the intercept and slope of the plot of $ln\ K_c$ versus $1/T$. C_{Ae} is adsorbed concentration of adsorbate over adsorbent and C_e is the adsorbate concentration in aqueous phase.

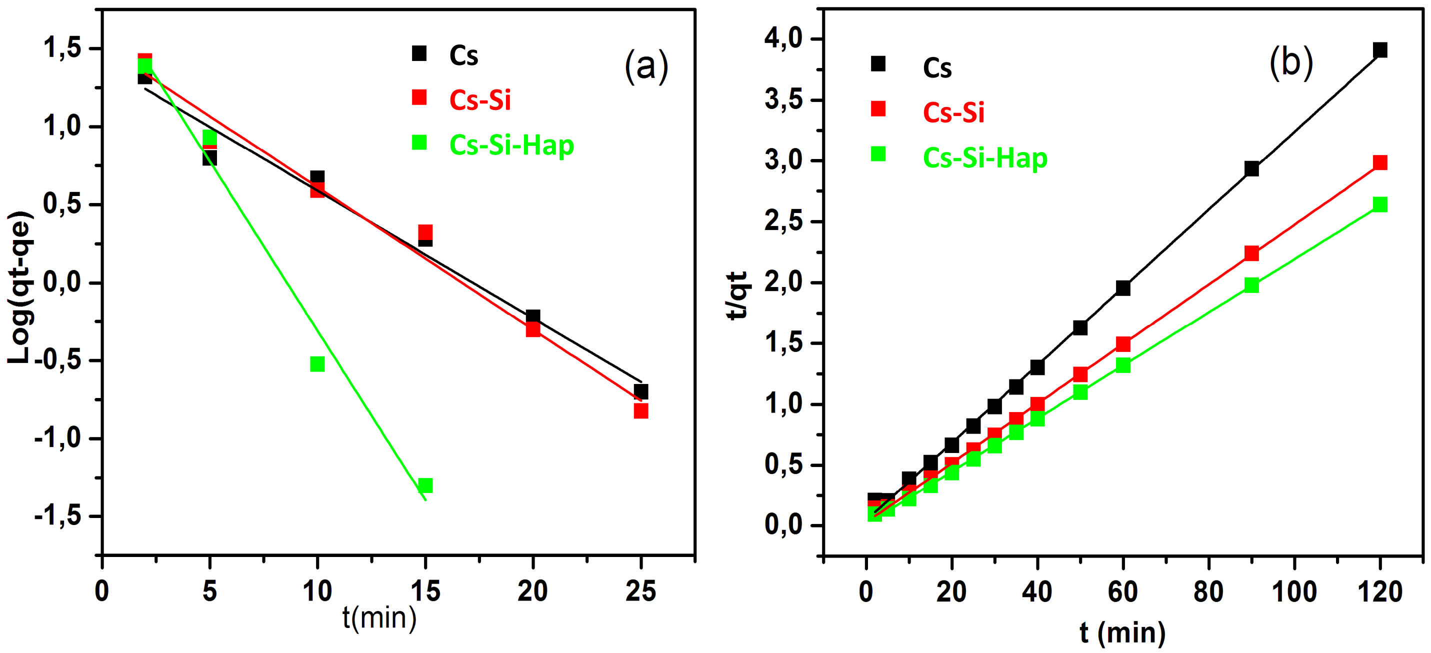

Figure S1 Linearized pseudo-first-order (a), and pseudo-second-order (b) kinetic plots for Cr(VI) adsorption. (Plotted in Origin software)

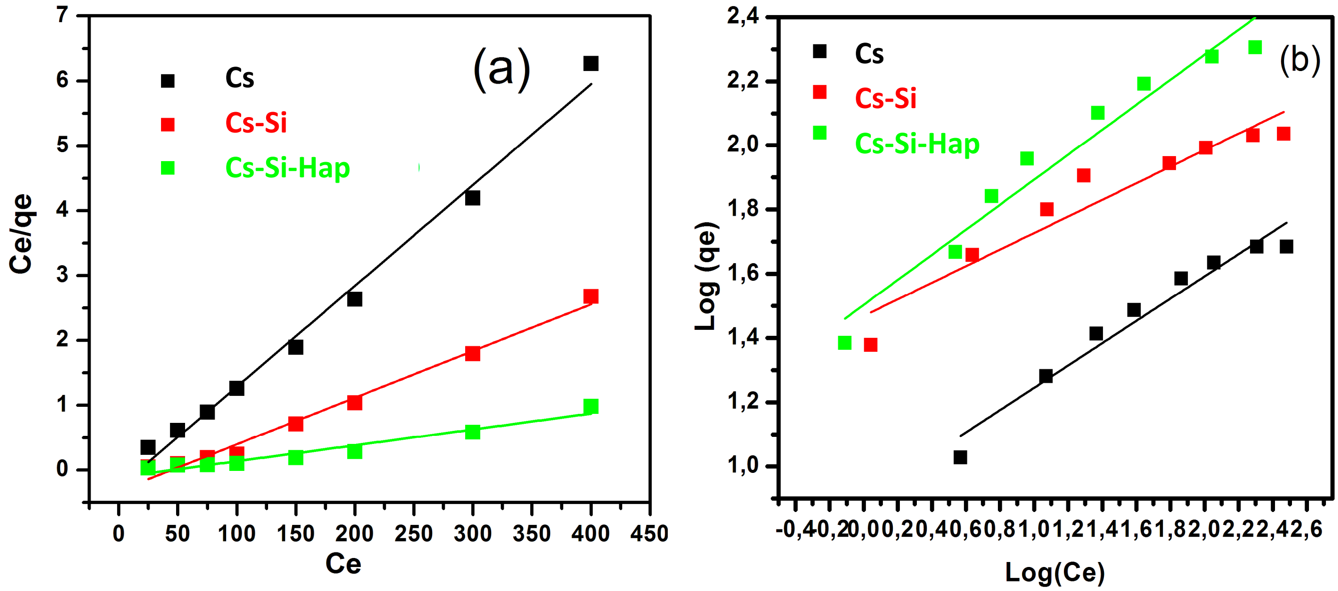

Figure S2 Linearized Langmuir **(a)**, and Freundlich **(b)** isotherm plots for Cr(VI) adsorption (Plotted in Origin software).

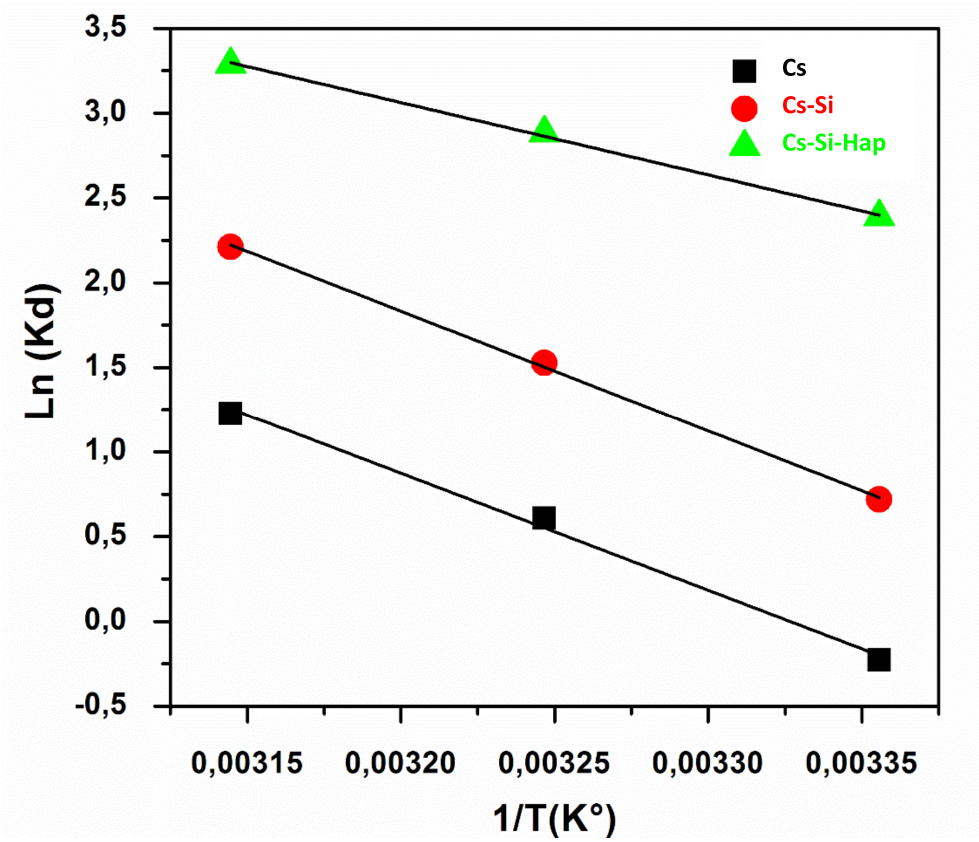

Figure S3 Van't Hoff's plots for Cr(VI) adsorption (Plotted in Origin software)
